# Multi-drug loaded micelles delivering chemotherapy and targeted therapies directed against HSP90 and the PI3K/AKT/mTOR pathway in prostate cancer

**DOI:** 10.1371/journal.pone.0174658

**Published:** 2017-03-28

**Authors:** Bao Le, Ginny L. Powers, Yu Tong Tam, Nicholas Schumacher, Rita L. Malinowski, Laura Steinke, Glen Kwon, Paul C. Marker

**Affiliations:** Division of Pharmaceutical Sciences, School of Pharmacy, University of Wisconsin-Madison, Madison, Wisconsin, United States of America; National Health Research Institutes, TAIWAN

## Abstract

**Background:**

Advanced prostate cancers that are resistant to all current therapies create a need for new therapeutic strategies. One recent innovative approach to cancer therapy is the simultaneous use of multiple FDA-approved drugs to target multiple pathways. A challenge for this approach is caused by the different solubility requirements of each individual drug, resulting in the need for a drug vehicle that is non-toxic and capable of carrying multiple water-insoluble antitumor drugs. Micelles have recently been shown to be new candidate drug solubilizers for anti cancer therapy.

**Methods:**

This study set out to examine the potential use of multi-drug loaded micelles for prostate cancer treatment in preclinical models including cell line and mouse models for prostate cancers with *Pten* deletions. Specifically antimitotic agent docetaxel, mTOR inhibitor rapamycin, and HSP90 inhibitor 17-N-allylamino-17-demethoxygeldanamycin were incorporated into the micelle system (DR17) and tested for antitumor efficacy.

**Results:**

*In vitro* growth inhibition of prostate cancer cells was greater when all three drugs were used in combination compared to each individual drug, and packaging the drugs into micelles enhanced the cytotoxic effects. At the molecular level DR17 targeted simultaneously several molecular signaling axes important in prostate cancer including androgen receptor, mTOR, and PI3K/AKT. In a mouse genetic model of prostate cancer, DR17 treatment decreased prostate weight, which was achieved by both increasing caspase-dependent cell death and decreasing cell proliferation. Similar effects were also observed when DR17 was administered to nude mice bearing prostate cancer cells xenografts.

**Conclusion:**

These results suggest that combining these three cancer drugs in multi-drug loaded micelles may be a promising strategy for prostate cancer therapy.

## Introduction

Recently there has been a growing interest in combination therapies that simultaneously target multiple important pathways in tumorigenesis and tumor progression. One factor that limits the potential for combination therapies is the combined toxicities from multiple drug delivery vehicles. On top of the classic toxicities associated with the mechanism of actions of chemotherapy, severe side effects caused by drug solubilizers are other hurdles of this traditional treatment. Peripheral neurotoxicity, hypersensitivity, adverse effects in gastrointestinal system, and ultimately life-threatening complications in a small subset of patients have been reported to be associated with Cremophor EL and polysorbate 80 (Tween 80) and DMSO/egg phospholipid, which are routinely used to formulate paclitaxel, rapamycin, and 17-N-allylamino-17-demethoxygeldanamycin (17-AAG) [1–3]. These adverse effects associated with drug solubilizers add to toxicity already increased in combination therapy, presenting a challenging hurdle for attempts to combine targeting agents for more efficacious cancer treatments. Poly(ethylene glycol)-*block*-poly(D,L-lactic actid) (PEG-*b*-PLA) micelles have gained increasing attention as drug delivery vehicles in preclinical drug development due to their nanoscopic dimensions, safety profile, and high capacity for solubilization of poorly water soluble drugs [4]. Given these superior properties of micelles, Shin et al. packaged three poorly water-soluble drugs, i.e. paclitaxel, rapamycin, and 17-AAG, in their micelles system, creating a nanocontainer for these drugs [5] that has been tested in *in vitro* and *in vivo* models for breast and lung cancer, and showed enhanced antitumor efficacy with lower toxicity responses [6].

Data from recent randomized trials of prostate cancer have suggested that docetaxel chemotherapy combined with androgen deprivation therapy promise an improvement in patient outcomes including overall survival and quality of life [7]. Given that PI3K/AKT/mTOR pathway and androgen receptor (AR) are highly relevant in the context of prostate cancer, and that there is a crosstalk between the PI3K/AKT/mTOR pathway and the AR axis [8], these findings suggest that extending the use of 3-in-1 drug system [5] to prostate cancer is worth exploring. To make the drug system more applicable to prostate cancer treatment, paclitaxel was replaced with docetaxel, a classic antimitotic first-line chemotherapy approved for prostate cancer [9], and the system will be referred as DR17.

In prostate cancer one of the most frequently mutated/deleted genes is the tumor suppressor gene phosphatase and tensin homolog (*Pten)*, a negative regulator of PI3K activity and thus AKT pathway [10]. The prostates from mice with prostate-specific *Pten* deletion (*Pten-Pb-Cre* mouse model) have been shown to progress through pathological stages similar to human prostate cancers including high-grade PIN (prostatic intraepithelial neoplasia), adenocarcinoma, and metastasis [11]. From the *Pten-Pb-Cre* mouse model, cell lines including PTEN-P2 (heterozygous for *Pten* deletion) and PTEN-CaP2 (homozygous for *Pten* deletion) were derived to generate models for *in vitro* studies [12]. The cytotoxic effects and changes in molecular signaling pathways upon the treatment of DR17 were evaluated in *in vitro* system using the PTEN-P2 and PTEN-CaP2 cell lines. Similar cytotoxic effects and molecular changes were also observed *in vivo* with DR17 treatment of the *Pten-Pb-Cre* mouse model and xenograft models of prostate cancer. These data complementarily showed the promising antitumor effects of DR17 in prostate cancer *in vitro* and *in vivo* preclinical models by simultaneously targeting both the PI3K/AKT/mTOR pathway and the AR axis.

## Materials and methods

### Cell lines and pharmacologic agents

Prostate cell lines PTEN-P2 and PTEN-CaP2 (courtesy of Dr. Hong Wu from UCLA) [12] were cultured in 4.5g/L glucose DMEM containing 10% heat inactivated fetal bovine serum, 1% penicillin/streptomycin, 6ng/mL human EGF, 5μg/mL insulin, and 25μg/mL bovine pituitary extract. 22Rv1 cells were purchased from the American Type Culture Collection (ATCC, Manassas, VA) and were cultured in RPMI 1640 (ATCC) supplemented with 10% heat inactivated fetal bovine serum, and 1% penicillin/streptomycin. The preparation of docetaxel, 17-AAG and rapamycin (LC laboratories, Woburn, WA) loaded PEG-*b*-PLA micelles was described in previous study [5]. The three-drug combination micelles were prepared as previously described [5] with weight ratio of 1:5:1 for docetaxel:17-AAG:rapamycin.

### Immunoblotting

After PTEN-P2 and PTEN-CaP2 reached approximately 80% confluence, they were treated with empty micelle control, docetaxel or rapamycin or 17-AAG loaded micelle, or DR17 for 24 hours. Cells were harvested for protein extraction using RIPA lysis buffer. Extracted proteins were quantified using BCA protein assay (Thermo Fisher Scientific Inc.) and were used for Western Blot following protocol as described in previous study [6]. Primary antibodies for Western blot detection were from Cell signaling Technology Inc. unless stated otherwise: p70S6K (1:1000), phospho-p70s6K (1:500), AKT (1:1000), phospho-AKT S473 (1:500), phospho-AKT T308 (1:500), HSP90 (1:1000), HSP70 (1:500), beta-actin (Sigma, 1:4000). Secondary antibodies (both from Santa Cruz Biotechnology) used for detection was either anti-rabbit IgG-HRP (1:10,000) or anti-mouse IgG-HRP (1:10,000). Blots were developed using enhanced chemiluminescence (ECL) detection system (Thermo Fisher Scientific Inc.).

### Immunohistochemistry

Immunohistochemistry (IHC) was performed as previously described [13] using antibody for Ki-67 (Abcam), alpha-smooth muscle actin (SMA) (Sigma), p63 (Biocare), cytokeratin (Dako), cleaved caspase-3, phospho-AKT, phospho-p70s6K (Cell signaling Technology Inc.), and AR (Santa Cruz). The number of positive cells for Ki-67 and p63 was counted to generate labeling indices using ImageJ (National Institute of Heath). Ki-67 data were presented as the percentages of positive cells (epithelial and stromal) to total number of cells (epithelial and stromal). For p63, data were the percentages of positively stained epithelial cells to total number of epithelial cells. For cleaved caspase 3, in *Pten* het tissue sections, positive staining of cleaved-caspase 3 was found in a single cell pattern. Therefore the data for *Pten* het were shown as the percentage of positive cells over total cells. In *Pten* null tissue sections, there were extensive areas where cleaved caspase 3-positively stained cells blended together, making it impossible to unambiguously count the number of positively stained single cells. As the consequence a modified approach was adopted for the assessment of positive staining in *Pten* null tissues, i.e. positively and negatively stained areas were determined using Photoshop, and quantified using ImageJ to give the final result of percentage of positively stained areas over total areas (sum of positive and negative areas).

### Cell growth and viability assays

Cell proliferation and viability were assessed via Alamar Blue assay (Life Technology) according to the manufacturer’s manual. Specifically PTEN-P2 and PTEN-CaP2 were plated at 250 cells/well in a 96 well plates 24 hours before the addition of conditioned media: DHT supplemented media for androgen response assay, and for drug assay: micelle loaded with single drug, micelle loaded DR17, free form DR17, or empty micelle. 72 hours after DHT supplement or drug treatment, Alamar Blue was added into cell cultures and fluorescence signal was read on a Fluostar Omega fluorescence plate reader (BMG Labtech) at 544/590 nm.

### Animal study

All experiments using mice were performed using protocols approved by the University of Wisconsin Institutional Animal Care and Use Committees. PB-Cre4 mice [14] and loxP-flanked Pten mice (*Pten*^*L/L*^*)* were used in the breeding scheme to generate *Pten*^*L/+*^*; PB-Cre4+* mice (heterozygous deletion of Pten, or Pten het) and *Pten*^*L/L*^*; PB-Cre4+* mice (homozygous deletion of Pten, or Pten null) as describe previously in Wang et al. [11]. Four-month old mice (16–17 weeks) were treated with DR17 loaded micelle or micelle empty control via tail vein injection once per week for 3 weeks. The administered dose of DR17 was 10mg/kg docetaxel, 50mg/kg 17-AAG, and 10mg/kg rapamycin, based on previously published study [6]. Mice were weekly weighed through the study and were sacrificed 2 days after the final injection. Tissues were dissected and formalin fixed for IHC for Ki-67, cleaved caspase-3, and prostate stromal and epithelial markers as described above. For the xenograft studies, 22Rv1 or CaP2 cells (8 x 10^5^) were combined with collagen mix (BD Bioscience) to make 20–25μL collagen pellets for xenograft surgery. Pellets were surgically grafted under the kidney capsules of male Balb/C nu/nu mice of 10 weeks of age (Charles River). Xenografts were grown *in vivo* for 4 weeks at which time mice were treated with DR17 loaded micelle or micelle empty control via tail vein injection with the mentioned dose once per week for 2 weeks. Two days after the second injection, mice were sacrificed for dissection and weight collection of the xenografts.

## Results

### *Pten* mutant prostate cancer cells have reduced growth in response to the increasing doses of docetaxel, 17-AGG, and rapamycin

**Fig 1:**
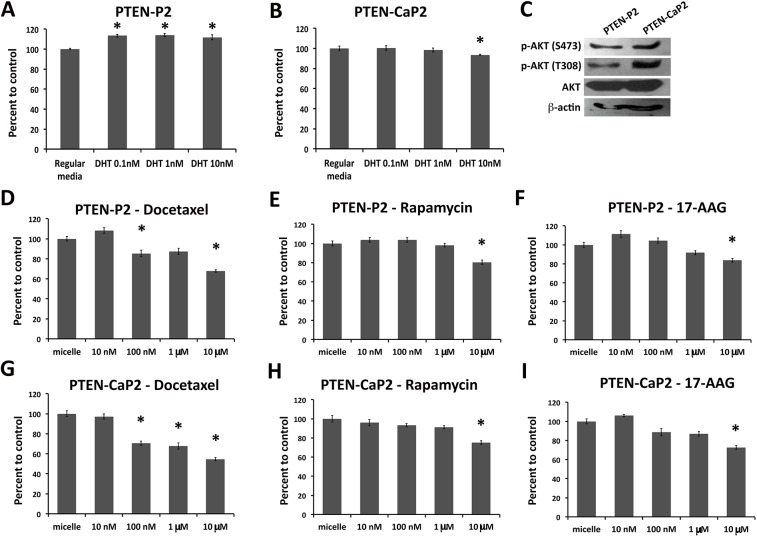
Dose response of prostate cancer cells to individual drugs. (A-B) Cells were grown in either control regular media or media supplemented with DHT of increasing concentrations. Cell growth was assessed using Alamar Blue assay as described in Materials and Methods. Results are representative of 3 independent experiments. (C) Immunoblotting was performed from equal amount of total protein using the phospho-AKT, AKT, and β-actin antibodies as described in Materials and Methods. (D-I) Cytotoxic effects of increasing doses of docetaxel (D, G), rapamycin (E, H), and 17-AAG (F, I) in PTEN-P2 cells (D, E, F) and PTEN-CaP2 cells (G, H, I). Cells were exposed to the indicated concentration of drug-loaded micelle or empty micelle for 72 hours. Cell viability was assessed using Alamar Blue assay as described in Materials and Methods. * indicates statistically significant differences compared to micelle control (ANOVA, p < 0.05). Results are representative of 3 independent experiments.

Directly derived from the *Pten-Pb-Cre* mouse prostate cancer model [12], the PTEN-P2 and PTEN-CaP2 cell lines were chosen as an initial *in vitro* prostate cancer model to test the efficacy of DR17 that targets the PTEN/PI3K/AKT and AR pathways. To look into the androgen response of PTEN-P2 and PTEN-CaP2, cells were grown in regular media or DHT supplemented media. The androgen effect on growth was quantified using Alamar Blue reagent. After 72 hours PTEN-P2 showed a modest but statistically significant growth induction compared to the control (Fig 1A). This androgen response was not observed in PTEN-CaP2 cells (Fig 1B). Since AKT signaling is one of the targets of DR17, AKT phosphorylation status was assessed in PTEN-P2 and PTEN-CaP2. Both cell lines had AKT phosphorylation at both sites S473 and T308, as shown by Western Blot, with PTEN-CaP2 had a slightly higher level of phospho-AKT (S473 and T308) compared to PTEN-P2 (Fig 1C). To examine the responsiveness of prostate cancer cells to the cytotoxic effects of single drug loaded micelles (docetaxel, rapamycin, 17-AAG), PTEN-P2 (Fig 1D, 1E and 1F) and PTEN-CaP2 (Fig 1G, 1H and 1I) were treated with increasing doses of single drug loaded micelle from 10nM to 10μM. The cytotoxic effect was quantified after 72 hours via cell viability Alamar Blue assay. PTEN-P2 and PTEN-CaP2 showed a decrease in the relative number of viable cells as the dose of drug increased from 10nM to 10μM. A dose response and significant inhibition of cell growth was observed in both cell lines and for all single drugs administered.

### *Pten* mutant prostate cancer cells showed reduced growth in response to increasing doses of DR17, and the cytotoxic effects of DR17 were higher than the effects of single drugs

**Fig 2:**
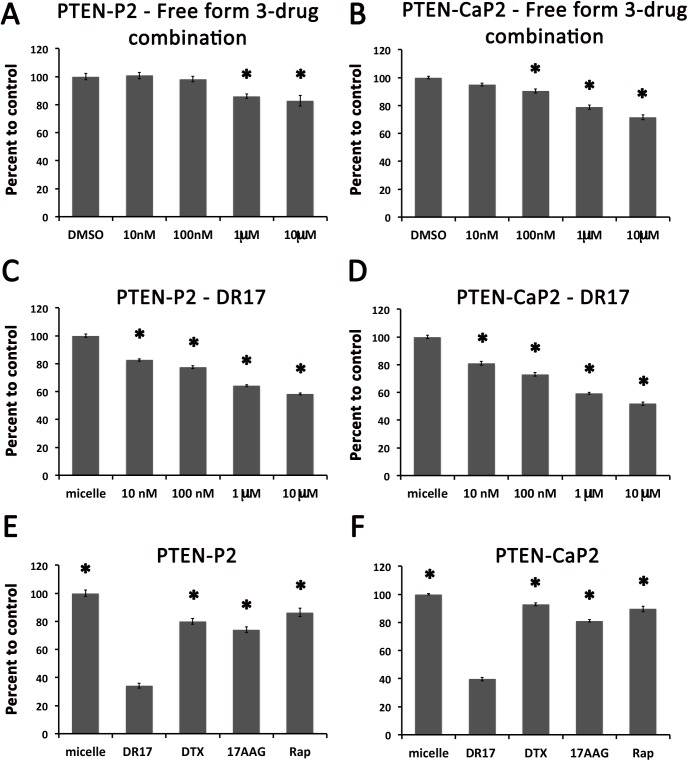
Dose response of prostate cancer cells to multi-drug loaded micelles. Cytotoxic effects of DR17 in PTEN-P2 (A, C, E) and PTEN-CaP2 cells (B, D, F). Dose responses of PTEN-P2 (A, C) and PTEN-CaP2 (B, D) to DR17 free form or DR17 delivered by micelle (DR17). Cells were exposed to the indicated concentrations of free form DR17 or DMSO, or DR17 loaded micelle or empty micelle for 72 hours. Cell viability was assessed using Alamar Blue assay as described in Materials and Methods. E-F, Comparison of cytotoxic effects of three-drug combination DR17 to individual drug loaded micelles (C, D). For C and D, DR17 was added to the media to the final concentration of 105μM. Final concentrations of docetaxel, rapamycin, 17-AAG were calculated based on molarity ratio of each individual drug in the DR17 formula [docetaxel:rapamycin:17-AAG (1:1:8.5)], which are 12.38μM, 10.94μM, and 85.37μM, respectively. Cells were exposed to the indicated drug conditions for 72 hours. Cell viability was assessed using Alamar Blue assay as described in Materials and Methods. * indicates statistically significant differences compared to DR17 treatment (ANOVA, p < 0.05). Results are representative of 3 independent experiments.

The cytotoxic effects of free form three-drug combination (docetaxel, rapamycin, and 17-AAG delivered by DMSO) and DR17 loaded micelles (DR17) were examined by treating PTEN-P2 and PTEN-CaP2 with increasing doses of DR17 or free form three-drug combination from 10nM to 10μM. After 72 hours post-treatment the cytotoxic effects were quantified by cell viability Alamar Blue assay (Fig 2). Both free form three-drug combination (Fig 2A and 2B) and DR17 delivered by micelles (Fig 2C and 2D) showed a significant dose-dependent cytotoxic effect on PTEN-P2 and PTEN-CaP2. The dose response of PTEN-P2 and PTEN-CaP2 to DR17 was noted to be more pronounced in DR17 loaded micelle treatment than in free form DR17, and also more robust compared to the response to individual drugs within the same range of dose treatment. Next the cytotoxicity effect of DR17 and single drugs were directly compared. The amount of DR17 used in our *in vitro* and *in vivo* studies were kept as the same drug concentrations that were optimized in the previous Triolimus study [6]. Twenty four hours after being plated, cells were treated with DR17 and cell viability was measured at 72 hours. DR17 showed a significantly more potent cytotoxicity on PTEN-P2 and PTEN-CaP2 compared to each individual drug delivered in micelles (Fig 2E and 2F). Both DR17 and single drug treatments had significant inhibitory effects on viable cell numbers compared to micelle treatment controls.

### Mechanism of action of DR17 at the level of cellular signaling

**Fig 3:**
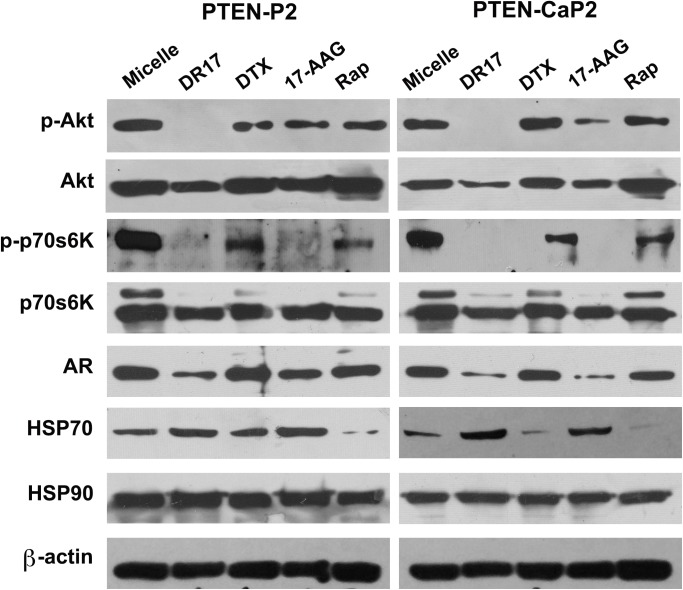
Expression of proteins in PI3K/Akt and AR pathways in PTEN-P2 and PTEN-CaP2 cells in response to DR17, docetaxel (DTX), 17AAG, and rapamycin (Rap) treatments. DR17, docetaxel, 17AAG, and rapamycin were added to media to the final concentration of 105μM, 12.38μM, 85.37μM, and 10.94μM, respectively, in which concentrations of individual drug were calculated based on their molarity ratio in DR17 formula [docetaxel:rapamycin:17-AAG (1:1:8.5)]. Cells were exposed to drug treatments for 24h, after which total protein was collected. Immunoblotting was performed from equal amount of total protein using the indicated antibodies as described in Materials and Methods. Results are representative of 3 independent experiments.

The molecular effects on signal transduction pathways targeted by individual drugs and DR17 in *in vitro* models were examined by Western blotting. In particular HSP70, HSP90, and AR protein levels were assessed as markers for the HSP70-HSP90-AR complex, phospho-p70s6k as marker for mTOR activation, and phospho-AKT as marker for PI3K/AKT activation. DR17 and 17-AAG treatments consistently resulted in the inhibition of p70s6K phosphorylation in both PTEN-P2 and PTEN-CaP2, whereas docetaxel and rapamycin treatment slightly decreased the level of p70s6k phosphorylation as shown in Fig 3. In terms of AKT activation, DR17 treatment led to the inhibition of AKT activation in both cell lines. 17-AAG and rapamycin single drug treatment also caused a slight decrease of AKT phosphorylation compared to basal level in micelle control samples. Treatment with DR17 and 17-AAG targeted HSP90 and caused a loss in AR protein in both cell lines upon treatment. An upregulation of HSP70 expression was observed upon treatment with DR17 and 17-AAG.

### DR17 reduced prostate and seminal vesicle weights in Pten-Pb-Cre mouse model and inhibited growth of prostate cancer xenografts, without causing systemic toxicity

**Fig 4:**
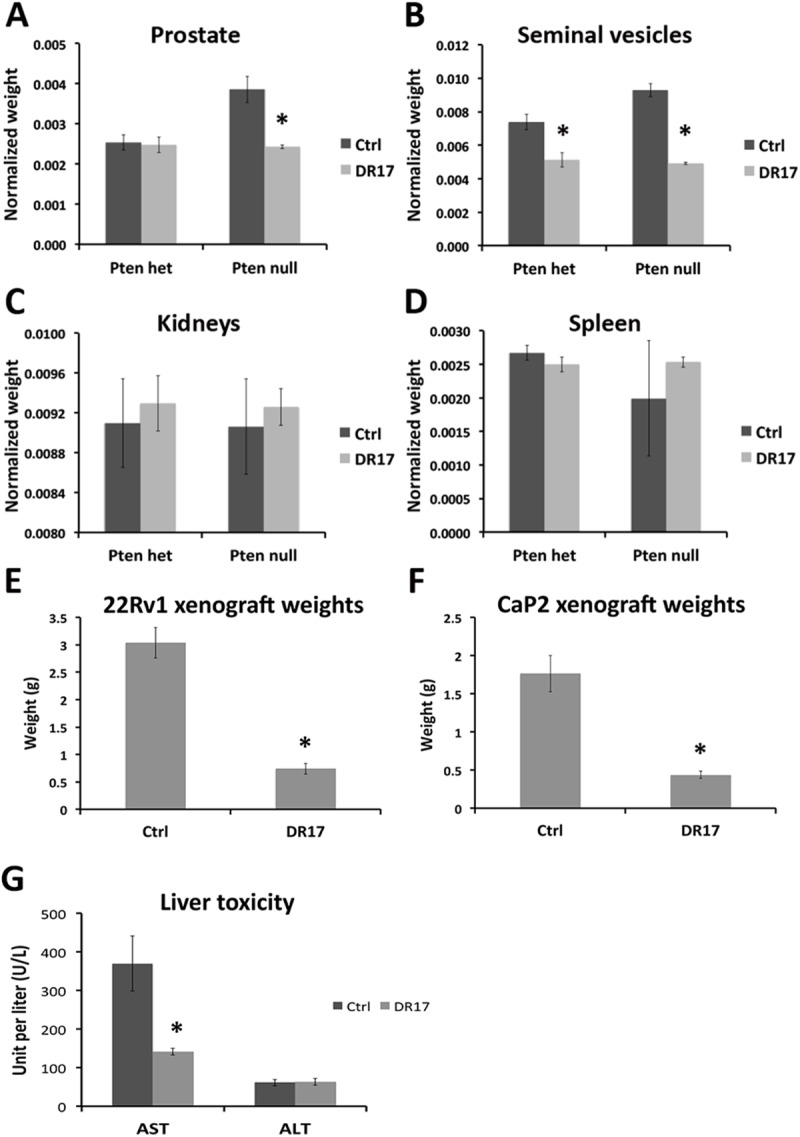
**Effect of DR17 on prostate (A), seminal vesicles (B), kidney (C), and spleen (D) in Pten-Pb-Cre mouse model of prostate cancer and in prostate cancer xenograft model (E-H).** Pten het (N = 9) and Pten null (N = 6) were treated with empty micelles (Ctrl) or DR17 loaded micelles for 3 weeks. Animals were sacrificed two days after the last injection, and organs were collected. The weights of all organs were normalized to total body weight. The weights of two kidneys were average and normalized to total body weight. Nude mice were grafted with 22Rv1 or CaP2 cell pellets for four weeks followed by 2 weeks of tail vein injection. Animals were sacrificed two day after the last injection. Blood samples were collected for liver toxicity assessment and the xenografts were collected and weighed. Dash lines indicate the normal range of AST and ALT level in nude mouse. * indicates statistically significant differences between DR17 and control group (p < 0.05).

In Pten-Pb-Cre mouse model of prostate cancer, significant reductions of prostate and seminal vesicle wet weights were observed in the DR17-treated group compared to the micelle control group in *Pten* null mice (Fig 4A and 4B). DR17 treatment also caused seminal vesicle weight reduction in *Pten* heterozygous mice (Fig 4B). The weights of kidney and spleen were collected as means to assess of systemic cytotoxicity, and there was no difference in the weights of kidney or spleen among the treatment groups (Fig 4C and 4D). 22Rv1 is another prostate cancer cell line included in this study due to its tumorigenic capability in xenograft model. In addition 22Rv1 also expresses mutated AR-V7, which was reported to be associated with castration-resistant prostate cancer [15]. Together with its expression of wild-type PTEN and AR, 22Rv1 provided another good model for castration resistant prostate cancer. When nude mice bearing 22Rv1 or CaP2 xenografts were treated with DR17, xenograft sizes were dramatically reduced (Fig 4E and 4F). Liver enzymes AST and ALT were measured as parameters of liver toxicity for both control and DR17 group of nude mice. As shown in Fig 4G, there was no difference in the level of ALT between the two groups of treatment, whereas the AST level was higher in the control group compared to DR17 group. The average ALT and AST level of control group and DR17 group were within the normal range of ALT and AST reported in nude mouse, normal range indicated by dashed lines in Fig 4G and 4H [16].

### DR17 did not cause dramatic changes in prostate cellular organization

**Fig 5:**
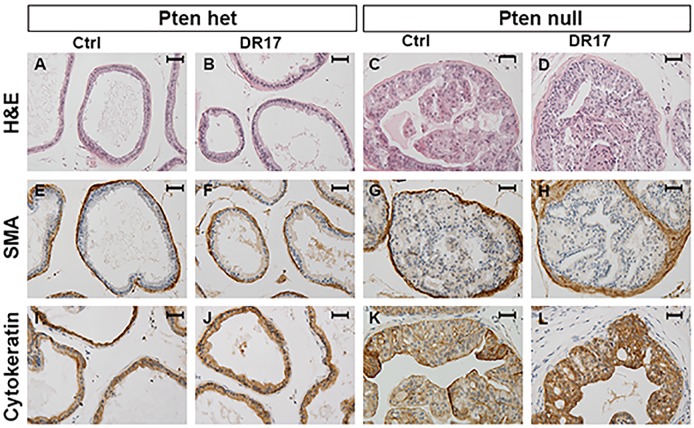
Histology of DR17 and micelle control treated prostates. Pten het N = 9 (A, B, E, F, I, J) and Pten null N = 6 (C, D, G, H, K, L) mice were treated with empty micelles (Ctrl) or DR17 loaded micelles for 3 weeks. Animals were sacrifice two days after the last injection. Prostates were dissected and fixed in formalin. Prostate sections were used for H&E staining to assess overall morphology (A-D). Immunohistochemistry was performed to examine stromal smooth muscle actin (SMA, E-H) and epithelial cytokeratin (I-L) markers. Pictures were taken at 40X magnification. The black bar in each picture represents 30 microns.

To determine the effects of DR17 on histology and organization of the prostate, hematoxylin and eosin (H&E) staining and IHC were performed on prostate sections of control and DR17 treated mice (Fig 5). Tissues of *Pten* heterozygous and *Pten* null mice appeared to have prostate histopathology as previously described [11], which was not changed by treatment with DR17, as shown in H&E staining (Fig 5A–5D). Next a closer examination of the effect of DR17 on prostate epithelium and stroma was conducted by IHC with stromal marker SMA (Fig 5E–5H) and epithelial cytokeratins (Fig 5I–5L). Staining of the stromal and epithelial compartment appeared to remain unchanged between the micelle control group and DR17 group. The presence and severity of high-grade PIN caused by *Pten* deletion was not significantly different in response to DR17 treatment as assessed by veterinary pathologist Ruth Sullivan.

### DR17 targeted phospho-AKT, phospho-p70s6K, and AR in prostate tissues

**Fig 6:**
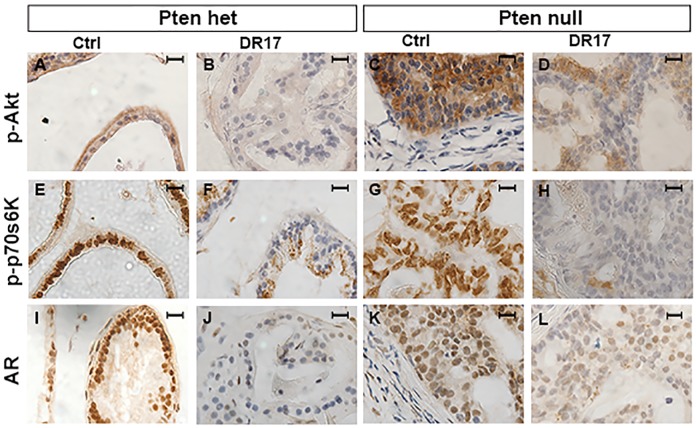
Phosphorylation levels of AKT and p70s6K and expression of AR in prostate tissues in response to DR17 treatment. Four-month old mice of two genotypes: PB-CRE4; Pten^loxp/+^ (Pten het) (N = 9) (A, B, E, F, I, J), and PB-CRE4; Pten^loxp/loxp^ (Pten null) (N = 6) (C, D, G, H, K, L) were treated with empty micelles or DR17 loaded micelles [docetaxel:rapamycin:17-AAG (10:10:50 mg/kg)] by weekly tail vein injection for 3 weeks. Animals were sacrificed two days after the last injection. Prostates were dissected and fixed in formalin. Immunohistochemistry staining for phospho-AKT (p-Akt) (A-D), phospho-p70s6K (p-p70s6K) (E-H), and AR (I-L) was performed on prostate tissue sections. Pictures were taken at 100X magnification. The black bar in each picture represents 10 microns.

The mechanism of the action of DR17 was examined in prostate tissues by IHC, specifically the phosphorylation of AKT and p70s6K, and the protein level of AR. DR17 treatment resulted in the inhibition of AKT (Fig 6A–6D) and p70s6K (Fig 6E–6H) phosphorylation compared to micelle control treatment in both *Pten* heterozygous and *Pten* null prostatic tissues. The protein level of AR also decreased in response to DR17 treatment compared to micelle control (Fig 6I–6L).

### DR17 increased prostatic apoptosis, decreased prostatic cell proliferation, and normalized the number of p63 positive cells in *Pten* null mice

**Fig 7:**
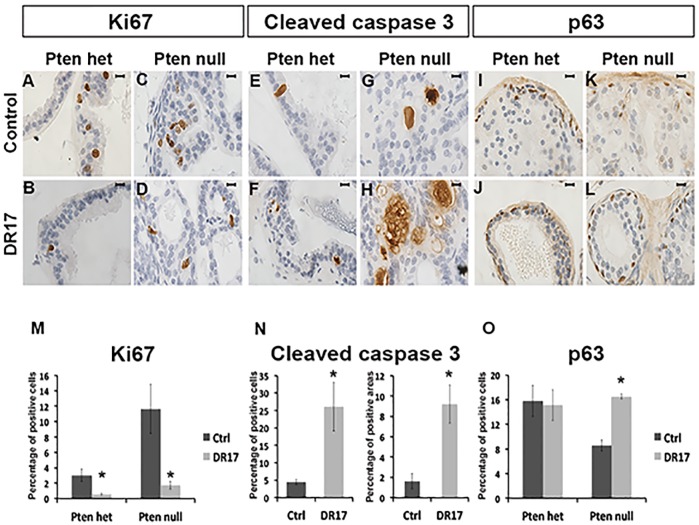
Effects of DR17 on cell proliferation, apoptosis, and prostate basal cells. Pten het N = 9 (A, B, E, F, I, J) and Pten null N = 6 (C, D, G, H, K, L) mice were treated with empty micelles or DR17 loaded micelles for 3 weeks. Animals were sacrifice two days after the last injection. Prostates were dissected and fixed in formalin. Immunohistochemistry staining for proliferation marker Ki67 (A-D), apoptosis marker cleaved caspase 3 (E-H), and basal cell marker p63 (I-L) was performed on prostate tissue sections. Pictures were taken at 100X magnification. The black bar in each picture represents 10 microns. Quantification of Ki67 (M), cleaved caspase 3 (N), and p63 (O) labeling index was conducted as described in Materials and Methods. Differences from control were statistically significant as indicated by * (Student’s *t* test, p < 0.05).

Treatment of DR17 led to a reduction of cell proliferation, assessed by Ki-67 (Fig 7A–7D), by more than 80% compared to micelle control treatment in both *Pten* heterozygous and *Pten* null prostatic tissues. (Fig 7M). DR17 treatment also resulted in an increase in the number and extent of cleaved-caspase 3 (an indicator of cell apoptosis) positive cells (Fig 7E–7H and 7N). In addition, there was an increase in the ratio of p63-positive basal cells to total epithelial cells in the prostates of the DR17-treated *Pten* null mice compared to the micelle-treated controls (Fig 7I–7L). Interestingly, the increase of p63-positive cell percentage in Pten null animals treated with DR17 brought the ratio of p63 positive basal cells versus total epithelial cells back to the level found in Pten heterozygous control mice (Fig 7O).

## Discussion

With the safety and efficacy of Triolimus having been confirmed *in vivo* in previous studies [5, 6], the focus of our study was to investigate the efficacy of DR17 and exploring its mechanism of action in prostate cancer using *in vitro* and *in vivo* models. In agreement with previous study of Triolimus which showed a higher efficacy of Triolimus compared to single drug treatment in breast and lung cancer *in vitro* models [6], DR17 exerted significantly more cytotoxic effects compared to individual drug loaded micelles at the doses found in the DR17 system in prostate cancer cell line models (Fig 2). Due to their low water solubility, docetaxel, rapamycin, and 17-AAG was dissolved in DMSO for delivery as three drug combination in our *in vitro* study, where we compared the efficacy of DR17 delivered by micelle and three drug combination delivered by DMSO. Our data showed that DR17 delivered by micelle exerted a stronger cytotoxic effect on PTEN-P2 and PTEN-CaP2 cells compared to three drug combination delivered by DMSO (Fig 2). It was however not possible to make this comparison *in vivo* since the volume of DMSO required for drug delivery of the three drug combination without micelles would be too large for an animal study. Together with our data from in vitro study showing that DR17 delivered by micelle exhibited a higher cytotoxic effect than the free form three-drug combination (Fig 2), the beneficial use of micelle as the delivery system for these drugs was well supported.

Our *in vitro* and *in vivo* studies also addressed the molecular mechanisms of DR17 cytotoxicity. Treating cells with 17-AAG or rapamycin loaded micelles resulted in a slight decrease in AKT activation (Fig 3). This observation was in agreement with the previous reports of the decrease of AKT phosphorylation in response to rapamycin treatment (via mTORC2 pathway) [17], and to the inhibition of HSP90 [18]. It has also been shown that docetaxel was able to induce AKT activation in prostate cancer cell lines and breast cancer cell lines [19, 20]. Therefore by combining docetaxel with the other two drugs targeting AKT signaling pathway the inhibition of AKT activation was achieved more efficiently with DR17, as seen in *in vitro* and *in vivo* results (Figs 3 and 6A–6D). PI3K/AKT/mTOR signaling pathway has complicated signaling regulation and feedback inhibition. mTOR activation acts as a negative feedback to regulate PI3K/AKT and MAPK/ERK signaling axes [21]. The antitumor effects from mTOR inhibition by rapamycin are often limited in animal studies and clinical trials, probably due to feedback reactivation [22]. In our *in vitro* and *in vivo* models the treatment of three-drug combination led to the significant inhibition of p70s6K and AKT phosphorylation (Figs 3 and 6A–6H), suggesting that the simultaneous targeting of both AKT and mTOR might be necessary to overcome the compensatory signaling and achieve the desirable antitumor effect.

Another signaling pathway targeted by DR17 is HSP90/HSP70/AR. 17-AAG targets HSP90, a molecular chaperone that plays a critical role in regulating many aspects of protein homeostasis. It has been showed that the binding of 17-AAG to HSP90 can induce the upregulation of HSP70 [23, 24], possibly contributing the mitigation of therapeutic effect of HSP90 inhibitor [25]. In agreement with reported studies, the inhibition of HSP90 by DR17 or 17-AAG resulted in an upregulation of HSP70 in both PTEN-P2 and PTEN-CaP2 cell lines (Fig 3). The targeting of 17-AAG to HSP90 also has a consequential effect on AR which is stabilized by HSP90 [26]. Our *in vitro* data indeed showed that 17-AAG treatment resulted in a decrease in AR expression in both PTEN-P2 and PTEN-CaP2. This effect on AR expression was observed at a greater extend in the treatment with DR17 (Figs 3 and 6I–6L), probably due to the complex cross-talk of AR and PI3K/AKT signaling pathway [27], suggesting that three-drug combination DR17 might provide a better targeting of AR and PI3K/AKT signaling pathway in prostate cancer.

From our *in vivo* study, a significant decrease in weight of the seminal vesicles in response to DR17 treatment was observed (Fig 4B). This could be explained by the targeting of HSP90 by DR17, hence indirectly inhibiting AR signaling. This result was to be expected as the function and histologic normality of seminal vesicles is highly sensitive to androgen and AR signaling. In mice, the seminal vesicles have been shown to atrophy upon castration [27]. In humans a decrease in size of seminal vesicle was also observed in patients receiving androgen ablation therapy [28]. In addition the treatment of DR17 resulted in a decrease of prostate weight in Pten null mice but not in Pten het mice (Fig 4A). This weight reduction was shown to be the consequence of both increased apoptosis (cleaved caspase 3) and decreased cell proliferation (Ki67) (Fig 7), in agreement with results from the Triolimus study [6]. Similar effects of DR17 have also been observed in prostate cancer xenograft models. Specifically DR17 treatment inhibited the growth of xenografts, shown as graft weights in Fig 4E and 4F. Data from our *in vivo* study also showed that there was no difference in the weights of the kidneys or spleens between control and DR17 groups (Fig 4C and 4D). When looking at liver toxicity parameters, there was no difference in the level of liver enzyme ALT between control and DR17. The difference in AST level between the control and DR17 group was suspected to be due to very large tumor burden in the control group. The average level of ALT and AST of both control and DR17 group still felt within the normal range of nude mice [16] (Fig 4G and 4H). All together data from our vivo studies using both mouse model and xenograft model positively support the delivery efficiency and treatment efficacy of the DR17 loaded micelle system without observable side effect toxicity, in agreement with data from previous study of Triolimus [6]. The significant efficacy of DR17 observed in both Pten-Pb-Cre mouse model and xenograft models of prostate cancer suggests that DR17 delivered by micelles can reach both the normal organ site (prostate) and the ectopic sites in xenografts. Lastly the efficacy of DR17 in inhibiting growth of graft in both 22Rv1 and CaP2 xenograft model implies that this three drug combination delivered by micelle can target PTEN-mutated prostate cancer as well as castration-resistant prostate cancer.

DR17 treatment did not dramatically alter the histology of the prostates in treated mice (Fig 5), and it did not eliminate the pathological pattern of PIN caused by *Pten* mutation. However it was promising that the treatment of *Pten* null prostates with DR17 normalized the ratio of p63-positive basal cells versus total epithelial cells (Fig 7O). The relative loss of basal cells is one of the hallmarks of PIN lesion and prostate cancer progression [6]. It has been shown that the majority of cancer cells in human prostate adenocarcinomas express markers similar to luminal but not basal cells [29]. More recently it has been suggested that luminal cells might play an important role in cancer progression [30]. Thus, even though PIN lesions were not eliminated by DR17 treatment, its effect on the ratio of p63-positive basal cells versus total epithelial cells might suggest selective killing of p63-negative, *Pten* mutant luminal epithelial cells. In summary data from our *in vivo* study showed that DR17 triggered desirable effects including reducing *Pten* null prostate weight and normalized the ratio of p63-positive basal cells versus total epithelial cells without causing systemic toxicity. Data from the *in vitro* study further suggested that the efficacy of DR17 treatment resulted, in part, from the targeting of multiple prostate cancer relevant signaling pathways including PI3K/AKT/mTOR and HSP90/HSP70/AR.

## Conclusion

There have been a number of studies adopting the approach of combining therapeutic agents to simultaneously target different oncogenic signaling pathways and achieve desirable antitumor effect in prostate cancer [31]. These studies mainly focused on the molecular effects on signaling pathways and the promising anticancer outcome. However, the practicality of delivering these therapeutic agents simultaneously in one delivery system that offers limited cytotoxic side effects *in vivo* models has yet to be addressed. The PEG-*b*-PLA micelle system that we used in our study provides an effective and safe solution to deliver multiple drugs simultaneously. We examined the efficacy of three drug combination, docetaxel, rapamycin, and 17-AAG, in prostate cancer *in vitro* and *in vivo* models, and confirmed the safety of the PEG-*b*-PLA micelle system and the higher efficacy of combining these therapeutic agents by simultaneous targeting multiple signaling pathways, in agreement with reported studies conducted in breast and lung cancer models [32]. Results from recent and ongoing clinical trials showed the survival benefits for prostate cancer patients when docetaxel is introduced in the treatment regimen early in the stage of hormone sensitive disease [5, 6]. Due to severe side effects come with chemotherapy, poor general health condition is one of the absolute contraindications for patients ineligible for docetaxel treatment [7, 33, 34]. The PEG-*b*-PLA micelle system with minimal side effects and high capability of delivering poorly water-soluble drugs offers a chemotherapeutic delivery system that allows the inclusion of prostate cancer patients with frail condition. PEG-*b*-PLA micelle also offers the possibility of combining of androgen deprivation therapy with chemotherapy and small molecular target therapy, promising the delay of cancer progression by simultaneously targeting of key oncogenic signaling pathways.
